# A genome- and phenome-wide association study of plasma procalcitonin concentrations in individuals of European ancestry

**DOI:** 10.1016/j.ebiom.2025.106014

**Published:** 2025-12-16

**Authors:** Wenbo Zhang, Peter J. van der Most, Siqi Wang, Zoha Kamali, Alice Giontella, Sofia Enhörning, Ron T. Gansevoort, Pim van der Harst, Stephan J.L. Bakker, Olle Melander, Frederik Keus, Gerton Lunter, Harold Snieder

**Affiliations:** a Department of Epidemiology, University of Groningen, University Medical Center Groningen, 9713 GZ, Groningen, the Netherlands; b Department of Critical Care, University of Groningen, University Medical Center Groningen, 9713 GZ, Groningen, the Netherlands; c Department of Bioinformatics, Isfahan University of Medical Sciences, Isfahan, 81746-73441, Iran; d Department of Clinical Sciences Malmö, Lund University, 205 02, Malmö, Sweden; e Perinatal and Cardiovascular Epidemiology, Department of Clinical Sciences Malmö, Lund University, 205 02, Malmö, Sweden; f Department of Internal Medicine, Skåne University Hospital, 205 02, Malmö, Sweden; g Division of Nephrology, Department of Internal Medicine, University of Groningen, University Medical Center Groningen, 9713 GZ, Groningen, the Netherlands; h Department of Cardiology, University Medical Center Groningen, University of Groningen, 9713 GZ, Groningen, the Netherlands; i Division of Heart & Lungs, Department of Cardiology, University Medical Center Utrecht, Utrecht, 3584 AA, the Netherlands; j Department of Clinical Sciences Malmö, Lund University, 21428, Malmö, Sweden; k Department of Internal Medicine, Skåne University Hospital Lund, 22242, Lund, Sweden

**Keywords:** Procalcitonin, Genome-wide association study, Phenome-wide association study, Expression quantitative trait loci

## Abstract

**Background:**

Procalcitonin (PCT) is a biomarker used to differentiate between viral and bacterial infections, though the underlying mechanisms are not yet fully understood. This study aimed to identify genetic variants associated with plasma PCT concentrations and explore the associations of genetically predicted PCT with a wide range of disease related traits in a PheWAS.

**Methods:**

We conducted GWAS and meta-analysis using data from the MDCS (*n* = 4007), MPP (*n* = 5097), and PREVEND (*n* = 3344) cohorts. We used fine-mapping to prioritise likely causal variants and explored regulatory effects using eQTL data, summary-data-based Mendelian randomisation (SMR) and colocalisation. To validate the PCT findings, we conducted multi-trait analysis of GWAS (MTAG) combining our results with *CALCA* data from a large pQTL study. The polygenic risk score (PRS) for PCT was calculated in the UK Biobank (*n* = 457,418) based on the GWAS summary data, and associations between the PRS and 179 traits were assessed in a PheWAS.

**Findings:**

We identified four independent significant SNPs in three loci associated with plasma PCT: *CALCB* (rs7119706, rs10832337), *PBX4* (rs17217098), and *PRDM15* (rs7277773). Fine-mapping prioritised 18 likely causal variants, including rs7119706 (near *CALCB*) and rs16930609 (mapped to *CYP2R1*) at the chromosome 11 locus. Our eQTL lookup identified significant results for 13 genes, but SMR and colocalisation analyses did not support their potentially causal effects on plasma PCT. The MTAG identified 28 additional significant SNPs across 14 loci. The PheWAS results revealed that PRS was associated with calcium metabolism-related traits, including calcium concentrations (*p* = 7.0 × 10^−5^), vitamin D concentrations (*p* = 2.0 × 10^−219^), and bone fractures (*p* = 6.5 × 10^−4^); metabolic traits, cardiovascular, renal, and liver function-related traits, and inflammation and immune-related traits.

**Interpretation:**

Our findings suggest that genetically predicted PCT is associated with multiple pathways including calcium metabolism and immune function, and has potential clinical implications for bone health, kidney function, and type 2 diabetes.

**Funding:**

China Scholarship Council (File no. 202006210041 to WZ and 201906010319 to SW, respectively).

**Research in context**

**Evidence before this study**

Procalcitonin (PCT) is widely used as a biomarker to differentiate bacterial from viral infections, yet its genetic determinants and broader biological implications remain unclear. To the best of our knowledge, no genome-wide association studies (GWASs) had previously been conducted to identify genetic variants associated with plasma PCT concentrations. Additionally, while PCT has been linked to various clinical conditions, there has been limited systematic investigation of its genetic associations with disease-related traits.

**Added value of this study**

We identified four independent genome-wide significant variants in three loci (*CALCB*, *PBX4*, *PRDM15*) associated with PCT levels. Post-GWAS analysis revealed that *ATP13A1* may have a causal effect on plasma PCT concentrations. Fine-mapping prioritised 18 variants with high posterior probability of causality, including rs7119706 and rs16930609 near *CALCB* and *CYP2R1*. Our eQTL lookup identified significant results for 13 genes, but SMR and colocalisation analyses did not support their potentially causal effects on plasma PCT. The MTAG identified 28 additional significant SNPs across 14 loci. The PheWAS showed that genetically predicted PCT was associated with calcium metabolism (e.g., vitamin D, bone fractures), metabolic traits (e.g., LDL cholesterol, T2D), cardiovascular and renal function (e.g., angina, eGFR), and immune-related traits (e.g., CRP).

**Implications of all the available evidence**

Our findings provide insights into the genetic architecture of PCT and its potential biological pathways. The associations between PCT and multiple traits highlight its broader physiological relevance beyond infection. These results underscore the need for further studies in clinical populations to explore PCT's role in disease pathophysiology and its potential for personalised medicine applications in critical care.

## Introduction

Procalcitonin (PCT), a precursor molecule to the hormone calcitonin (CT),^1^ is recognised as a biomarker that holds promise in distinguishing between viral and bacterial infections.^2^ PCT is typically used in combination with other clinical tests to diagnose the type and severity of infections, guide decisions on reducing and discontinuing antibiotic therapy, and evaluate patient prognosis.^3^^,^^4^ Additionally, researchers have shown that PCT concentrations are associated with heart failure in patients with bacterial infection,^5^ ulcerative colitis,^6^ and diabetic ketoacidosis complicated by pancreatitis.^7^

Despite its clinical significance, no genome-wide association study (GWAS) has yet been conducted on PCT concentrations. The genetic variants influencing PCT concentrations and the mechanisms underlying its associations with multiple diseases remain unclear. To address this, we conducted a GWAS meta-analysis in over 12,000 Europeans from three different cohorts. Additionally, we conducted a phenome-wide association study (PheWAS) to agnostically explore associations of the PCT polygenic risk score (PRS) and its individual genetic variants with a wide variety of disease related outcomes.

To date, few studies have focused on the genetic architecture of PCT plasma concentration variations. Here, we aimed to identify specific genetic variants associated with plasma PCT concentrations and explore the implications of its genetic architecture on diseases and related traits.

## Methods

### Ethics

The two Swedish cohorts “Malmö Diet and Cancer study” (MDCS) and “Malmö Preventive Project” (MPP) have ethics approval from the Regional Ethics Committee at the University of Lund (LU 51–90, LU 244-02, and Dnr 2009/633). The Dutch cohort “Prevention of REnal and Vascular ENd-stage Disease” (PREVEND) has ethics approval from the Medical Ethics Committee (MEC 96/01/022) of the University Medical Center Groningen. The UK Biobank (UKB) has ethics approval from the North West Multi-Centre Research Ethics Committee, the National Information Governance Board for Health & Social Care in England and Wales, and the Community Health Index Advisory Group for Scotland (REC reference: 16/NW/0274). All the studies were conducted in accordance with the Declaration of Helsinki, and all participants have provided informed consent.

### Population and study design

We included participants from three cohorts for the GWAS meta-analysis: the Malmö Diet and Cancer study (MDCS) and the Malmö Preventive Project (MPP), both consisting of participants from the city of Malmö in southern Sweden; the Prevention of REnal and Vascular ENd-stage Disease (PREVEND) study, consisting of participants from the city of Groningen in northern Netherlands; and conducted the PheWAS in the UK Biobank (UKB) from the United Kingdom.

MDCS is a population-based cohort of 30,447 participants who at an age of between 45 and 73 years underwent a health examination between 1991 and 1996. MPP is also population-based, including 33,346 participants recruited by pre-specified birth year groups for health examinations from 1974 to 1992. PREVEND is a population-based cohort with 8592 participants enrolled between 1997 and 1998. The UKB is a publicly available research resource, consisting of a population-based cohort of 502,627 men and women aged between 40 and 69 years, recruited nationally between 2006 and 2011. Detailed descriptions of all four cohorts are published elsewhere.^8^, ^9^, ^10^, ^11^

**Fig. 1:**
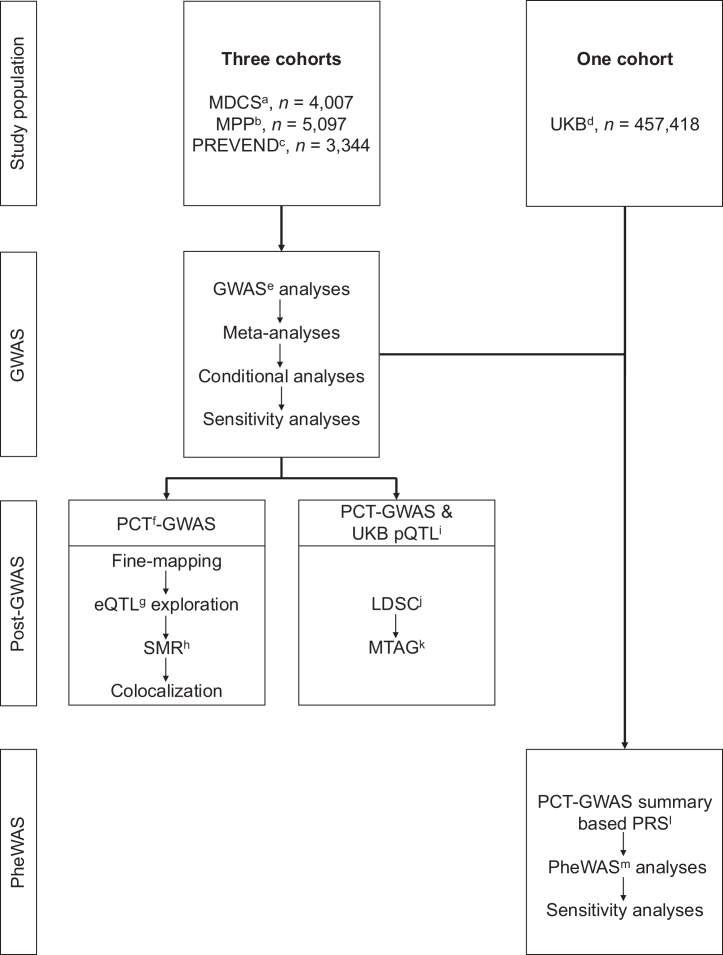
Schematic overview of the study design. a. MDC = Malmö Diet and Cancer cohort study; b. MPP = Malmö Preventive Project cohort; c. PREVEND = Prevention of REnal and Vascular ENd-stage Disease cohort; d. UKB= UK Biobank; e. GWAS = Genome-wide association study; f. PCT = Procalcitonin; g. eQTL = Expression quantitative trait loci; h. SMR = summary-data-based Mendelian randomisation; i. pQTL = Protein quantitative trait loci; j. LDSC = LD score regression; k. MTAG = Multi-trait analysis of GWAS; l. PRS = Polygenic risk score; m. PheWAS = Phenome-wide association study.

Following a natural log-transformation of the PCT data, we used the Kolmogorov–Smirnov test to confirm its normal distribution in three cohorts (Figure S1). Participants with missing data or with natural log-transformed plasma PCT concentrations beyond 4 standard deviations (SD) from the mean were excluded. Ultimately, we used data from 12,448 unrelated individuals of European ancestry with PCT measurements in the MDCS (n = 4007), MPP (n = 5097), and PREVEND (n = 3344) cohorts to conduct three GWAS studies, followed by a meta-analysis of all the GWAS results. For the post-GWAS analysis, the significant SNPs were checked in expression quantitative trait loci (eQTL) data from 49 tissues and cells to explore potential regulatory effects. Additionally, summary-data-based Mendelian randomisation (SMR) was performed to identify which eQTL genes had a causal effect on plasma PCT concentrations. Fine-mapping and colocalisation analyses were conducted to identify likely causal variants and determine whether associations with PCT and gene expression were driven by the same variants. We calculated PCT-PRS in 457,418 European participants in the UKB and conducted a PheWAS to investigate the association between the PCT-PRS and 179 traits. The overall study design and characteristics of each cohort are shown in Fig. 1 and Table 1 respectively.

**Table 1:** Characteristics of included participants in all four cohorts.

	MDCS *n* = 4007	MPP *n* = 5097	PREVEND *n* = 3344	UKBB *n* = 457,418
Age, *yrs*	57.7 (5.9)	69.4 (6.2)	49.2 (12.3)	57.3 (8.0)
Male, *n*	1679 (42.0)	3561 (70.0)	1679 (50.2)	209,035 (45.7)
Body index mass, *kg/m*^*2*^	25.9 (4.0)	27.2 (4.2)	26.1 (4.3)	27.4 (4.8)
Procalcitonin, *μg/L*	0.016 (0.013–0.021)	0.035 (0.026–0.047)	0.016 (0.013–0.020)	NA

Numbers are *n* (%), mean (SD) or median (interquartile range).

### PCT measurements

PCT was measured by a commercially available immunoluminometric assay (BRAHMS PCT sensitive LIA; Hennigsdorf, Germany) in MDCS, MPP, and PREVEND. Assays were performed in EDTA-plasma aliquots that had been stored frozen at −80 °C, without previous thawing and refreezing. The intraassay CV at 0.1 ng/ml was 6%, and at 0.03 ng/ml it was 8%. The functional assay sensitivity, defined as the lowest concentration to be determined with an interassay CV of 20% was 0.007 ng/ml. The lowest detection limit was 0.006 ng/ml.^12^ The assay technique has been described previously.^13^ All technicians were blinded to the participants’ characteristics.

### Genotyping and imputation

Genotyping was performed using the Illumina GWAS Chip (GSA v1 array) in the MDCS and MPP cohort. The MDCS and MPP genotype data was imputed using the Michigan Imputation Server with the Haplotype Reference Consortium (HRC r1.1) reference panel. The PREVEND genotyping was performed using the Illumina HumanCytoSNP12 v2 beadchip array (Illumina, Inc; San Diego, CA, USA). Genotypes were called with the Illumina GenomeStudio software package (Illumina, Inc). Details on genotyping and quality control (QC) have been described before.^14^ In the UKB, Affymetrix (ThermoFisher Scientifics) performed genotype calling on two closely related, but custom-designed arrays. Approximately 50,000 participants were run on the UK BiLEVE Axiom array and the remaining 450,000 were run on the UK-Biobank Axiom array. A detailed description of the genotype process and internal quality control is described elsewhere.^15^ The UKB data were imputed to a reference panel that merged the UK10K and 1000 Genomes Phase 3 reference panels, which consisted of 87,696,888 bi-allelic markers in 12,570 haplotypes.^15^

### GWAS and meta-analysis

*GWAS on plasma PCT concentrations* To harmonise the analysis we applied the same SNP exclusion criteria across the three cohorts, including low minor allele frequency (MAF) < 0.01, Hardy–Weinberg Equilibrium *p* < 1 × 10^−6^, and call rate <95%. GWAS was conducted with the PLINK 1.9 genome association analysis toolset.^16^ We applied a linear regression model for the natural log-transformed PCT concentrations and the SNPs from chromosomes 1 to 22 in the MDCS, MPP, and PREVEND cohorts. We adjusted for sex, age, body mass index (BMI), and the first ten principal components (PCs). It has been found that BMI is associated with PCT concentrations.^12^ Therefore, we adjusted for BMI in our primary GWAS analysis. However, it is well known that adjusting for traits that themselves have a considerable genetic component^17^ such as BMI might introduce collider bias.^18^ Therefore, we also conducted our GWASs without adjusting for BMI in sensitivity analyses. As for infection and immune-related diseases that may potentially affect PCT levels (Table S1), we constructed a linear model based on phenotypic data (Table S2) in the PREVEND cohort. However, since infection and immune-related diseases were not significantly associated with lnPCT levels, this variable was not included as a covariate in the GWAS analysis. Genome-wide statistical significance was established at a *p* < 5 × 10^−8^ for the meta-analysis results. Post-GWAS quality control was performed using the same *p* threshold, with the *GWASInspector* R package (v.1.6.4.0).^19^^,^^20^ The 1000 Genomes Project reference panel^21^ was used as a QC reference for allele frequency and constructed harmonised identifiers for meta-analyses. We annotated SNPs with rs accession numbers by using the *MungeSumstats* (v.1.10.1)^22^ and SNPannotator (v.0.2.6.0)^23^ R packages.

*GWAS meta-analysis* Inverse variance-weighted fixed-effects meta-analysis of all SNPs from our three cohorts was performed using METAL software.^24^ As with the previous QC steps, we used the *GWASInspector* R package to ensure standardisation and conduct QC on the post-meta-analysis summary data. Significant loci were mapped by using the Functional Mapping and Annotation of GWAS platform (FUMA).^25^ We applied the following formula to estimate the proportion of phenotypic variance explained by the combination of independent significant SNPs identified in the GWAS meta-analysis^26^:

$$\left(\sum_{i=1}^{n}2f_{i}\left(1-f_{i}\right)\beta_{i}^{2})/var(PCT\right)$$

where *f*i is the MAF of the *i*-th SNP; *β* is the effect size in the GWAS meta-analysis summary data of the *i*-th SNP; *n* is the total number of independent significant SNPs; *var* (PCT) is the average variance of natural log transformed PCT of the three cohorts. The meta-analysis results were used for all downstream analyses.

*Conditional analysis* We performed a conditional analysis with the PLINK 1.9 software to identify independent significant SNPs. We included each lead SNP as a covariate in the model of each GWAS cohort, considering a region within ±2 Mb around the SNP. We used LDlink to assess the linkage disequilibrium (LD) between any newly found significant SNP and the lead SNP.^27^

### Post-GWAS analysis

*Fine-mapping of causal variants* We used FINEMAP^28^ to identify likely causal variants at PCT GWAS loci. We included all SNPs within ±1 Mb of each of the four index SNPs. Z-files were constructed for each region, and LD matrices were generated using the 1000 Genomes Phase 3 European reference panel.^21^ FINEMAP v1.4.2 was then run with default settings to prioritise likely causal variants in each region.

*Investigating the association of PCT loci with gene expression levels* To explore the regulatory consequences of PCT loci, we searched significant SNP-gene expression associations, with false discovery rate (FDR) adjusted *p-values*<0.05, through lookups of the four lead SNPs in expression quantitative trait loci (eQTL) datasets of all 49 tissues and cells from the Genotype-Tissue Expression (GTEx) database v8.^29^ Genes that have an associated eQTL result are commonly called ‘eGenes’.^30^

*Identifying causal eGenes through Mendelian randomisation* We performed Summary-data-based Mendelian Randomisation (SMR),^31^ which is an adapted MR approach for eQTL data, to assess potential causal effect of eGenes on plasma PCT concentrations, using the largest available eQTL database (ñ32,000) in whole blood^32^ to maximise the power of detection. We used the 1000 genome reference panel^21^ for LD calculations. As per default QC process in SMR, SNPs with allele frequency difference >0.2 among pairs of input datasets (i.e., GWAS, eQTL, and the reference panel) were excluded from the SMR analysis. We set a Bonferroni corrected *p* threshold of 0.05/n_eGenes_ to filter SMR significant results. Given that LD between the distinct GWAS and eQTL SNPs might bias the MR estimate, we also applied the HEterogeneity In Dependent Instruments (HEIDI) test^31^ to flag significant results possibly confounded by pleiotropy.

*Colocalisation analysis with eQTL signals* To further investigate the potential causal relationship between gene expression and plasma PCT levels, we conducted colocalisation analysis on the SMR-significant eGenes. We used the COLOC R package^33^ to assess whether GWAS and eQTL signals colocalize, indicating a shared causal variant. We extracted SNPs within ±1 Mb of the gene and intersected them with GWAS results to perform colocalisation analysis.

*Cross-trait meta-analysis with CALCA pQTL data* PCT is encoded by the calcitonin-related polypeptide α (*CALCA*) gene,^34^ *CALCA* and *CALCB* have overlapping biological functions.^35^, ^36^, ^37^ To validate our GWAS findings, we obtained protein quantitative trait loci (pQTL) summary statistics of CALCA and CALCB from a large proteomics GWAS.^38^ Genomic build conversion was performed using LiftOver,^39^ and regional association plots for the CALCA and CALCB loci were generated with LocusZoom.^40^ We applied LD Score Regression (LDSC)^41^ to quantify the genetic correlation (r_g_) between PCT and these protein(s). We subsequently performed a multi-trait analysis of GWAS (MTAG)^42^ of PCT with the protein(s) showing a significant genetic correlation with PCT. We used FUMA for further analysis of the new summary results.

### PheWAS

*Trait selection* In our PheWAS we explored associations with a wide range of health-related traits in the UKB, including a total of 89 continuous and 90 binary traits. The list of continuous traits is presented in Table S3 and includes body measurements, blood and urinary biomarkers. The binary traits related to diseases and treatment were generated by using the R package *ukbpheno*,^43^ their definitions (shown in Table S4) are based on ICD9, ICD10, OPCS4, Self-reported data fields, READ2, and CTV3 codes. For disease outcomes, cases occurred either before inclusion or during follow-up (until March 31, 2021, for participants from England and Scotland; until February 28, 2018, for participants from Wales). This study was conducted under application number 74395, utilising data obtained from the UK Biobank resource.

*PheWAS analysis* We used the SBayesRC (R package v.0.2.3),^44^ a method that integrates functional genomic annotations with high-density SNPs (>7 million), for polygenic risk score (PRS) construction. Using our PCT GWAS summary data (adjusted for age, sex, BMI, and the first ten principal components as covariates), we calculated the PCT-PRS in 457,418 European individuals from the UKB. In our analyses, we used the PRS as well as the single genome-wide significant lead SNPs from our GWAS results as a genetic proxy of PCT. The PRS was standardised (z-scored) prior to regression modelling. Effect sizes from PheWAS analyses are interpreted per 1 SD increase in the PRS. Linear regression analyses were conducted to test the association between continuous traits and PCT-PRS or single SNPs. Traits were rank-based inverse normalised if the data deviated from normality. Outliers of continuous traits beyond 5 SD from the mean were excluded. We performed logistic regression analyses for binary outcomes with a prevalence over 1%. All regression analyses were adjusted for the covariates age, sex, genotyping chip, and the first 30 genetic PCs. A sensitivity analysis using 385,160 unrelated individuals was conducted to investigate the influence of familial relatedness. To account for multiple testing, we applied the FDR correction method, considering an FDR<0.05 as statistically significant. Regression analyses were performed using STATA version 16.^45^

### Role of funders

The funders had no role in the study design, data collection, analysis, interpretation of data, decision to publish, or preparation of the manuscript.

## Results

### GWAS and meta-analysis

**Fig. 2:**
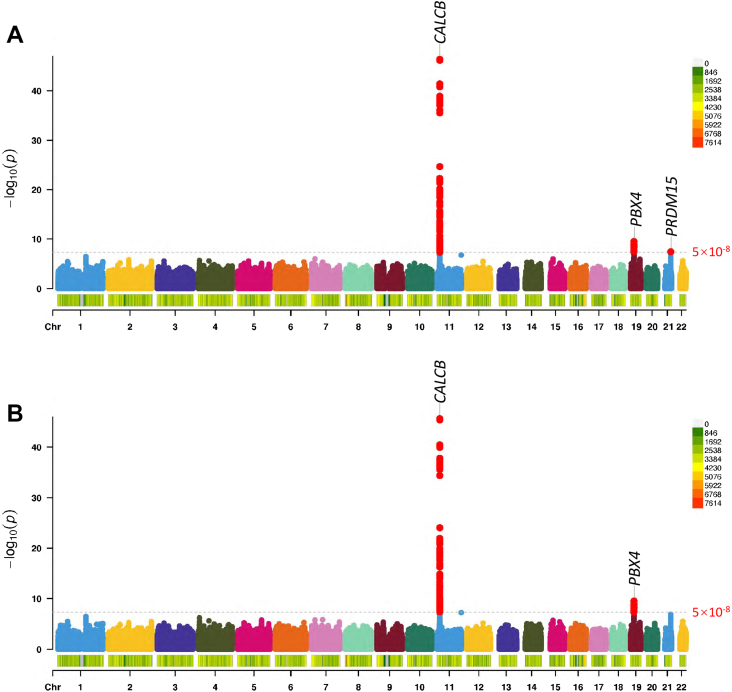
Manhattan plot of the meta-GWAS results. Model A: PCT ∼ age + sex + BMI + 10PCs. Model B (sensitivity analysis): PCT ∼ age + sex + 10PCs.

The study design is illustrated in Fig. 1. General characteristics of the MDCS (*n* = 4007), MPP (*n* = 5097), and PREVEND (*n* = 3344) cohorts are described in Table 1. In the meta-analysis, the top genome-wide significant (*p*-value threshold <5 × 10^−8^) hits for each locus were rs7119706 (beta = −0.065, *p* = 4.2 × 10^−47^, Wald test) on chromosome 11 at the *CALCB* locus, rs17217098 (beta = 0.050, *p* = 3.2 × 10^−10^, Wald test) on chromosome 19 at the *PBX4* locus, and rs7277773 (beta = −0.027, *p* = 3.8 × 10^−8^, Wald test) on chromosome 21 at the *PRDM15* locus. Details of the top associations are shown in Table 2 and Fig. 2. The phenotypic variance explained by the four independently significant SNPs was 1.8%.

**Table 2:** Independent top hits in the GWAS meta-analysis adjusted for BMI.

SNP	Chromosome	Position	Region	Nearest gene	Effect allele/other allele	Meta-analysis		
						EAF	Beta (95% CI)	*p*-value
rs7119706	11	14932145	Intronic	*CALCB*	A/G	0.45	−0.065 (−0.074, −0.056)	4.2 × 10^−47^
rs10832337^a^	11	14981266	Intronic	*CALCB*	A/G	0.33	0.031 (0.021, 0.041)	5.0 × 10^−9^
rs17217098	19	19702384	Intronic	*PBX4*	A/G	0.09	0.050 (0.034, 0.065)	3.2 × 10^−10^
rs7277773	21	43286000	Intronic	*PRDM15*	T/C	0.30	−0.027 (−0.037, −0.018)	3.8 × 10^−8^

EAF = effect allele frequency.

a Result of conditional analysis, when adjusting for rs7119706 as a covariate. The pairwise linkage disequilibrium with rs7119706 was r^2^ = 0.11.

In the sensitivity analysis without adjustment for BMI, rs7119706 and rs17217098 remained significant (beta = −0.065, *p* = 2.3 × 10^−46^ and beta = 0.051, *p* = 3.1 × 10^−10^ respectively, Wald test), but rs7277773 was no longer significant (beta = −0.026, *p* = 1.3 × 10^−7^, Wald test). In addition, there were two SNPs that were suggestive (*p* < 5 × 10^−7^) and exhibited low LD with the top hits. These were rs4129267 (beta = 0.023, *p* = 3.6 × 10^−7^, Wald test) on chromosome 1 at the *IL6R* locus, and rs76246580 (beta = −0.125, *p* = 1.74 × 10^−7^, Wald test) on chromosome 11 at the *OPCML* locus. More information can be found in Table S5. The effects of each SNP were consistent in different analyses (Figure S2).

We performed conditional analyses within the ±2 Mb regions around rs7119706 (chromosome 11, 14932145bp), rs17217098 (chromosome 19, 19702384bp), and rs7277773 (chromosome 21, 43286000bp). The results indicated the presence of only one additional independently significant SNP, rs10832337, located at the *CALCB* locus (*p* = 8.5 × 10^−9^, Wald test) (Table 2). The pairwise LD with rs7119706 was *r*^2^ = 0.18.

### Post-GWAS analysis

*Fine-mapping prioritises likely causal variants* Using FINEMAP, we identified 18 variants with >95% posterior probability of being likely causal across the four index SNPs of the three significant loci (Tables S6 and S7). In the chromosome 11 locus, both index SNPs (rs7119706 and rs10832337) highlighted rs16930609 (mapped to *CYP2R1*) as a likely shared causal variant. Additionally, the lead SNP rs7119706 (near *CALCB*) itself was a likely causal variant in the fine-mapping results of the secondary significant SNP rs10832337.

*Association of PCT loci with gene expression levels* Three out of the four PCT lead SNPs (rs7119706, rs17217098, and rs7277773) showed at least one significant (FDR-adjusted *p-values*<0.05) association with gene expression levels in GTEx tissues or cells. With 24 associations, rs17217098 was the most pleiotropic locus. Thirteen eGenes were found, out of which *ATP13A1,* with 16 associations in lung, skin, thyroid and other tissues, was the most frequently seen eGene. Fibroblast cells showed the highest number of associations in the results. For the tissues, the highest number of associations were found in blood, brain, tibial nerve, testis, and thyroid (Table S8).

*eGenes passed M. randomisation* The thirteen eGenes identified in the eQTL lookup were taken forward for SMR tests. Eleven of the 13 eGenes were present in the GTEx database v8 and six showed significant associations with plasma PCT concentrations through SMR analysis (*p* < 4.6 × 10^−3^, Bonferroni corrected *p*-value threshold): *PDE3B*, *CYP2R1*, *ATP13A1*, *COPB1*, *RRAS2*, and *MAU2*. However, none of these passed the strict HEIDI test threshold (*p*-HEIDI ≥0.05), although with a *p* of 0.012, *ATP13A1* showed only a modest departure from the null hypothesis, indicating some support for a potential causal effect (Table S9).

*Colocalisation analysis of the eGene ATP13A1* Colocalisation results (Table S10) provided limited support for a shared causal variant between *ATP13A1* expression and PCT concentration. There was only a 2.9% probability that the causal variant was shared. In contrast, there was a 97% probability supporting the presence of distinct causal variants for the gene expression and PCT levels.

In the regional comparison (Figure S3), the lead SNP from the *CALCA* pQTL results on chromosome 11, rs782744846, was mapped to *CALCB*, aligning with our PCT GWAS finding that rs7119706 was mapped to *CALCB*. These two SNPs are in perfect LD (*r*^2^ = 1.0). In the cross-trait LDSC analysis, PCT showed a significant genetic correlation with the CALCA protein (*r*_*g*_ = 0.91, nominal *p*-value = 0.005, Z-test), but not with *CALCB* (*r*_*g*_ = 0.18, *p* = 0.41, Z-test)). By applying MTAG, we increased the equivalent sample size of the PCT GWAS to 98,978, leading to the identification of 28 additional significant SNPs across 14 loci (Table S11). Notably, the new lead SNP on chromosome 11 near the *CALCA*/*CALCB* region (rs10766197) was in high LD with our original lead SNP rs7119706 (*r*^2^ = 0.94) and still mapped to *CALCB*, rather than *CALCA*. A similar pattern was observed on chromosome 19, where rs58542926 (mapped to *TM6SF2*) was in high LD with the original lead SNP rs17217098 (*r*^2^ = 0.72). Meanwhile, the previously identified SNP on chromosome 21 (rs7277773) was no longer significant in the MTAG analysis; however, its genetic association with PCT remained modest (*p* = 0.005, Wald test). In total, 13 loci were newly found to be significantly associated with PCT in the MTAG summary statistics (Figure S4).

### PheWAS

The PCT-PRS calculated in the 457,418 UKB participants was found to be associated with 46 different traits with FDR-adjusted *p*-values<0.05 (Table 3). The PCT-PRS showed significant associations with calcium metabolism including both higher calcium (beta = 5.8 × 10^−4^, se = 1.5 × 10^−4^, *p* = 7.0 × 10^−5^, Wald test) and vitamin D concentrations (beta = 0.049, se = 1.5 × 10^−3^, *p* = 2.0 × 10^−219^, Wald test), with vitamin D being the most significant trait among the 179 traits. Additionally, a higher PCT-PRS was associated with an increased risk of bone fractures (OR = 1.01, 95% CI 1.01–1.02, *p* = 6.5 × 10^−4^, Wald test).

**Table 3:** Significant PheWAS associations (false discovery rate <0.05) between the polygenic risk scores of procalcitonin and outcome traits in the UK biobank.

Trait	Beta	se	*p*	FDR
Vitamin D^a^	0.049	1.5 × 10^−3^	2.0 × 10^−219^	3.5 × 10^−217^
CRP^a^	0.019	1.5 × 10^−3^	5.6 × 10^−35^	4.0 × 10^−33^
Cystatin C^a^	0.017	1.4 × 10^−3^	6.7 × 10^−35^	4.0 × 10^−33^
Creatinine^a^	0.013	1.2 × 10^−3^	1.1 × 10^−26^	4.8 × 10^−25^
eGFR in CKD patients (mL/min/1.73 m^2^)	−0.168	0.017	1.6 × 10^−23^	5.8 × 10^−22^
ALT^a^	0.011	1.4 × 10^−3^	1.3 × 10^−14^	3.7 × 10^−13^
Platelet count	0.579	0.086	1.4 × 10^−11^	3.5 × 10^−10^
Platelet crit	4.6 × 10^−4^	6.9 × 10^−5^	1.6 × 10^−11^	3.5 × 10^−10^
Cholesterol (g/dl)	−0.428	0.066	9.1 × 10^−11^	1.8 × 10^−9^
Gamma GT^a^	9.2 × 10^−3^	1.4 × 10^−3^	9.9 × 10^−11^	1.8 × 10^−9^
LDL/Apolipoprotein B ratio	−8.5 × 10^−4^	1.4 × 10^−4^	1.9 × 10^−9^	3.1 × 10^−8^
LDL (mg/dl)	−0.29	0.051	1.4 × 10^−8^	2.0 × 10^−7^
Neutrophill (%)	8.6 × 10^−3^	1.5 × 10^−3^	1.5 × 10^−8^	2.1 × 10^−7^
WBC^a^	7.6 × 10^−3^	1.5 × 10^−3^	4.3 × 10^−7^	5.5 × 10^−6^
Direct bilirubin^a^	8.0 × 10^−3^	1.6 × 10^−3^	5.8 × 10^−7^	6.9 × 10^−6^
Maximum education years (yrs)^a^	−6.7 × 10^−3^	1.5 × 10^−3^	5.0 × 10^−6^	5.6 × 10^−5^
Lymphocyte (%)	−0.048	0.011	8.8 × 10^−6^	8.8 × 10^−5^
Total bilirubin^a^	6 5 × 10^−3^	1.5 × 10^−3^	8.7 × 10^−6^	8.8 × 10^−6^
Albumin (g/l)	0.018	4.1 × 10^−3^	1.6 × 10^−5^	1.5 × 10^−4^
Red blood cell count^a^	−6.2 × 10^−3^	1.5 × 10^−3^	3.4 × 10^−5^	3.0 × 10^−4^
Haemoglobin concentration (g/dl)	6.0 × 10^−3^	1.5 × 10^−3^	5.0 × 10^−5^	4.2 × 10^−4^
Neutrophill (%)^a^	0.050	0.012	5.8 × 10^−5^	4.7 × 10^−4^
Apolipoprotein B (g/l)	−1.5 × 10^−3^	3.6 × 10^−4^	7.2 × 10^−5^	5.0 × 10^−4^
Calcium (mmol/l)	5.8 × 10^−4^	1.5 × 10^−4^	7.0 × 10^−5^	5.0 × 10^−4^
Lean body mass^a^	−3.7 × 10^−3^	9.4 × 10^−4^	7.3 × 10^−5^	5.0 × 10^−4^
Triglyceride^a^	−5.9 × 10^−3^	1.5 × 10^−3^	6.8 × 10^−5^	5.0 × 10^−4^
Resting heart rate	−0.065	0.017	9.1 × 10^−5^	5.8 × 10^−4^
HbA1c^a^	5.6 × 10^−3^	1.5 × 10^−3^	1.3 × 10^−4^	7.8 × 10^−4^
Type 2 diabetes	0.022	5.8 × 10^−3^	1.6 × 10^−4^	9.5 × 10^−4^
Diabetes mellitus (medication included)	0.019	5.2 × 10^−3^	2.9 × 10^−4^	1.7 × 10^−3^
Bone fracture	0.012	3.6 × 10^−3^	6.5 × 10^−4^	3.7 × 10^−3^
Eosinphill (%)^a^	5.2 × 10^−3^	1.5 × 10^−3^	6.7 × 10^−4^	3.7 × 10^−3^
Predicted 24-h urinary potassium excretion (mg/day)^a^	−4.8 × 10^−3^	1.5 × 10^−3^	1.3 × 10^−3^	7.1 × 10^−3^
BSA (m^2^)	−7.7 × 10^−4^	2.5 × 10^−4^	1.7 × 10^−3^	8.8 × 10^−3^
Reticulocyte count	2.8 × 10^−3^	9.0 × 10^−4^	2.2 × 10^−3^	1.1 × 10^−2^
Pulse pressure	0.051	0.018	3.7 × 10^−3^	1.8 × 10^−2^
Red blood cell count	1.5 × 10^−3^	5.3 × 10^−4^	4.2 × 10^−3^	2.0 × 10^−2^
AST^a^	4.2 × 10^−3^	1.5 × 10^−3^	4.3 × 10^−3^	2.0 × 10^−2^
Apolipoprotein B/A1 ratio	−8.4 × 10^−4^	3.2 × 10^−4^	7.78 × 10^−3^	3.4 × 10^−2^
Diabetes-related eye disease	0.034	0.013	7.8 × 10^−3^	3.4 × 10^−2^
Reticulocyte (%)	2.3 × 10^−3^	8.8 × 10^−4^	9.1 × 10^−3^	3.9 × 10^−2^
Angina	0.015	5.8 × 10^−3^	9.7 × 10^−3^	4.0 × 10^−2^
Position of pulse wave notch	−0.041	0.016	1.1 × 10^−2^	4.7 × 10^−2^
Cancer (non-malignant)	−9.3 × 10^−3^	3.7 × 10^−3^	1.2 × 10^−2^	4.7 × 10^−2^
Injury	7.8 × 10^−3^	3.1 × 10^−3^	1.2 × 10^−2^	4.7 × 10^−2^

FDR = Benjamini & Hochberg False-discovery-rate corrected *p*-value; CRP = C-reactive protein; eGFR = estimated glomerular filtration rate; CKD = chronic kidney disease; ALT = alanine aminotransferase; Gamma GT = gamma-glutamyltransferase; LDL = low-density lipoprotein; WBC = white blood cell count; HbA1c = glycated haemoglobin; BSA = body surface area; AST = aspartate transaminase.

a Continuous traits that deviated from normality were transformed using rank-based inverse normalisation before association testing.

In terms of metabolic traits, the PCT-PRS showed significant associations with lower LDL cholesterol (beta = −0.29, se = 0.051, *p* = 1.4 × 10^−8^, Wald test), total cholesterol (beta = −0.43, se = 0.066, *p* = 9.1 × 10^−11^, Wald test), and increased risk of type 2 diabetes (OR = 1.02, 95% CI 1.01–1.03, *p* = 1.6 × 10^−4^, Wald test).

Cardiovascular, renal, and liver function markers were also linked to PCT-PRS, including angina (OR = 1.02, 95% CI 1.00–1.03, *p* = 9.7 × 10^−3^, Wald test), estimated glomerular filtration rate (eGFR) (beta = −0.17, se = 0.017, *p* = 1.6 × 10^−23^, Wald test), and alanine aminotransferase (ALT) (beta = 0.011, se = 1.4 × 10^−3^, *p* = 1.3 × 10^−14^, Wald test).

Furthermore, the PCT-PRS was associated with inflammation and immune-related traits, such as C-reactive protein (CRP) (beta = 0.019, se = 1.5 × 10^−3^, *p* = 5.6 × 10^−35^, Wald test), and haematological traits including platelet count (beta = 0.58, se = 0.086, *p* = 1.4 × 10^−11^, Wald test), suggesting a broad genetic influence of PCT across multiple physiological systems.

In the sensitivity analysis using only unrelated individuals (n = 385,160), the results were similar to the main analysis, with five traits (angina, aspartate aminotransferase, reticulocyte percentage, cancer (non−malignant), and injury) no longer significant. Full details were presented in Table S12. Regarding individual SNP results, rs17217098 (*CALCB*) was significantly associated with 68 traits, 38 of which were consistent with the PCT-PRS results, such as vitamin D, lean body mass, and bone fracture. For the other three SNPs, only 13, 8, and 3 traits were significantly associated, respectively. More details can be found in Table S13.

## Discussion

To date, few studies have focused on the genetic architecture of PCT plasma concentration variations. In the present study of 12,448 individuals of European descent, we identified three loci (*CALCB*, *PBX4*, *PRDM15*) and four independent variants associated with plasma PCT concentrations. The PCT-PRS, based on the GWAS results, was associated with various traits, including calcium metabolism-related traits (calcium, vitamin D), metabolic traits (total cholesterol, HbA1c, T2D), inflammation and immune-related traits (CRP, WBC). It was also associated with cardiovascular, renal and liver function-related traits, such as pulse pressure, resting heart rate, creatinine, cystatin C, eGFR, albumin, and bilirubin.

Previous studies have confirmed that PCT is encoded by *CALCA* located on chromosome 11.^34^ However, in our GWAS and validation results, we did not find any significant SNPs in *CALCA* associated with PCT concentrations. Instead, four SNPs from *CALCB*, *PBX4*, and *PRDM15* were associated with PCT concentrations.

In a previous candidate-gene study, Schoe et al. found significant associations between genetic variation in *CALCA* (rs3781719, *p* < 5 × 10^−4^, and rs2132466, *p* = 1 × 10^−4^) and PCT levels in 677 patients undergoing elective cardiac surgery.^46^ However, these SNPs were not significant in our GWAS results (*p* = 0.3 and 0.6 respectively). Thus, in a healthy population, *CALCA* may not significantly affect PCT levels. We believe this discrepancy could be due to their study not accounting for population structure or multiple testing corrections. Additionally, their participants were post-surgery patients likely to be more prone to infection, whereas our participants came from the general population. In the general population, PCT is produced by thyroid C cells, where it is cleaved into CT and secreted into the bloodstream. However, during bacterial infections, PCT is produced by adipocytes throughout the body, leading to a rapid increase in PCT levels.^47^ This explains why PCT levels in healthy individuals remain relatively low.^48^ To conclude, our GWAS findings were not entirely consistent with the CALCA pQTL analysis in the UK Biobank.^38^ However, we found that the reported lead SNP rs782744846 associated with CALCA protein levels (i.e., *CALCA* gene-related peptide (CGRP)) also mapped to *CALCB*, instead of *CALCA* and is in perfect LD with our own lead SNP from the PCT GWAS (rs7119706). This discrepancy likely arises due to the overlapping nature of the *CALCA* and *CALCB* genomic regions, which can lead to differences in gene annotation across platforms and genome builds. Moreover, although CGRP and PCT both originate from the *CALCA* gene, they are distinct peptides produced via alternative splicing and under different regulatory contexts. CGRP is expressed in neuronal tissues,^49^ whereas PCT is mainly produced in the thyroid.^50^ These biological and tissue-specific differences may explain the divergence in genetic association signals observed across datasets. Although *CALCB* does not encode PCT directly, *CALCA* and *CALCB* are paralogs, i.e., separated by gene duplication events, and have similar functions.^35^, ^36^, ^37^ However, it remains unclear why we find genome-wide significant signals in *CALCB* rather than *CALCA*, and further research is needed to provide precise explanations.

As for the other significant loci identified in our GWAS, *PBX4* is a transcription factor involved in various developmental processes and the regulation of immune responses.^51^^,^^52^ *PRDM15* is a transcription factor known for its roles in differentiation and gene regulation.^53^ Both genes are associated with general pathways involving metabolism, inflammation, and immune responses.

As mentioned earlier, PCT is a precursor molecule to the hormone CT which is secreted by the C-cells of the thyroid grand. CT plays a key role in regulating calcium metabolism, which involves several important organs, including the thyroid, kidneys, and bones.^54^^,^^55^

In the post-GWAS analysis, fine-mapping prioritised rs7119706—a variant near *CALCB*—with strong evidence of causality, along with rs16930609 mapped to *CYP2R1*. Meanwhile, SMR suggested a potential causal relationship between *ATP13A1* and plasma PCT. However, this was not supported by colocalisation analysis, which indicated that the association may be driven by LD rather than a shared causal variant. The MTAG results indicated that despite the genetic correlation with the CALCA protein, the regulatory variants influencing PCT levels may be more closely linked to the *CALCB* locus. Together, these results support a potential regulatory role of variants near *CALCB* and *CYP2R1* in influencing plasma PCT levels.

In the PheWAS analysis, the results revealed significant associations between PCT-PRS and several biomarkers related to bone metabolism, including vitamin D and calcium. Vitamin D is essential for calcium absorption and bone health.^37^ The strong association between PCT-PRS and vitamin D highlights the role of PCT in calcium-related metabolic pathways. Furthermore, the potential impact of PCT on vitamin D may indirectly influence calcium homoeostasis and bone mineralisation. Notably, the significant association between PCT-PRS and bone fractures underscores the clinical implications of potential dysregulation within these pathways.

In addition to calcium metabolism, PCT-PRS were significantly associated with renal function biomarkers, such as cystatin C, and creatinine. Since the kidneys play a crucial role in calcium reabsorption within renal tubules to maintain calcium balance,^56^ these associations could suggest pathways through which PCT impacts both calcium regulation and renal function.

Moreover, PCT-PRS showed associations with metabolic, cardiovascular, liver function, and immune-related traits. These potentially causal effects were also evident in single-SNP PheWAS results, particularly for rs17217098. There may be a potential positive causal relationship between PCT and neutrophil percentage, both of which exhibit distinct responses to bacterial and viral infections.^57^ This suggests that during bacterial infection, PCT and neutrophils may share similar metabolic pathways or mechanisms of interaction. The above-mentioned results show that PCT may be involved in multiple complex metabolic networks.

From a clinical perspective, many of the significant traits identified in the PheWAS—such as ALT, cystatin C, CRP, eGFR, and vitamin D—have previously been shown to be significantly associated with plasma PCT concentrations in patient populations.^58^, ^59^, ^60^, ^61^, ^62^, ^63^, ^64^, ^65^ However, it is important to note that those studies were conducted in patients, where infection and inflammation statuses differ from those in our general population cohort.

In the PheWAS analysis, the PCT-PRS serves as a genetic instrument to investigate potential causal links between PCT and other traits, applying principles similar to a one-sample Mendelian randomisation (MR) framework. However, the current approach cannot evaluate pleiotropy or apply sensitivity analyses less sensitive to violations of MR assumptions.^66^ Therefore, further validation of specific PheWAS results would require follow up with methods like latent causal variable modelling^67^ or two-sample MR.^66^

While our PheWAS identified a range of significant associations between the PCT-PRS and various traits, it is important to acknowledge that most effect sizes were modest. This is consistent with the polygenic nature of complex traits and the relatively small proportion of phenotypic variance explained by the PCT-PRS. As such, these findings are unlikely to have immediate predictive utility in clinical settings. Instead, their value lies in revealing shared genetic architecture and potential biological pathways linking procalcitonin regulation with metabolic, immune, and organ function–related traits. These insights can inform future mechanistic studies and hypothesis-driven research.

Our GWAS, post-GWAS, and PheWAS findings suggest potential biological mechanisms. The identified loci—*CALCB*, *PBX4*, *PRDM15*, and *ATP13A1*—may play roles in different metabolic pathways of this trait, contributing to a broader network involved in calcium metabolism, inflammation and immune responses, as well as cardiovascular, renal, and liver functions.

This study provides a comprehensive GWAS and PheWAS of plasma PCT concentrations. By including a relatively large sample size from multiple cohorts, we had sufficient power to identify loci associated with plasma PCT concentrations and explore traits potentially influenced by PCT.

However, several limitations should be noted. First, our study involved only northern European populations, so caution is needed when generalising the findings to other ethnicities. Second, we studied the general population rather than patients. PCT production varies between normal conditions and during infection or inflammation. Future studies focussing on patient populations are necessary to provide a more complete understanding of PCT metabolism across different contexts. Third, the context-dependent nature of gene expression, especially for immune traits like PCT, necessitates further research on eQTL function across physiological and pathological states and would, for example, require PCT measurements in response to acute infections.

Our findings suggest that genetic variants associated with PCT are associated with multiple metabolic and immune-related pathways. These results highlight the complex biological networks underlying PCT regulation.

## Contributors

HS, SJLB, GL, and FK proposed the idea. WZ, SW, and ZK performed the analyses. WZ wrote the first draft of the manuscript. WZ, PJvdM, SW, ZK, GL, FK, and HS interpreted results. PvdH, AG, SE, OM, RTG, and SJLB contributed to cohort data collection. WZ, SW, and ZK verified the underlying data. All authors read and approved the final version of the manuscript.

## Data sharing statement

Full GWAS summary data of our meta-analyses are publicly available at the GWAS Catalogue website (https://www.ebi.ac.uk/gwas) with data accession codes GCST90501352 and GCST90501353. Additional data used and/or analysed during the study are available from the corresponding author upon reasonable request.

## Declaration of interests

All authors declare that they have no conflicts of interest.
