# Supplementary material for: A genome- and phenome-wide association study of plasma procalcitonin concentrations in individuals of European ancestry
Source: eBioMedicine. 2025 Dec 16;123:106014. doi: 10.1016/j.ebiom.2025.106014 (PMC12902255; doi:10.1016/j.ebiom.2025.106014)
Supplement: Supplementary Figures S1–S4 [file mmc1.docx]

**Figure S1.** Histogram and Q-Q plots of natural log-transformed procalcitonin levels in three cohorts.

A. Histogram Plot of the natural log transformed procalcitonin levels in the cohort MDC

B. Q-Q Plot of the natural log transformed procalcitonin levels in the cohort MDC

C. Histogram Plot of the natural log transformed procalcitonin levels in the cohort MPP

D. Q-Q Plot of the natural log transformed procalcitonin levels in the cohort MPP

E. Histogram Plot of the natural log transformed procalcitonin levels in the cohort PREVEND

F. Q-Q Plot of the natural log transformed procalcitonin levels in the cohort PREVEND

**
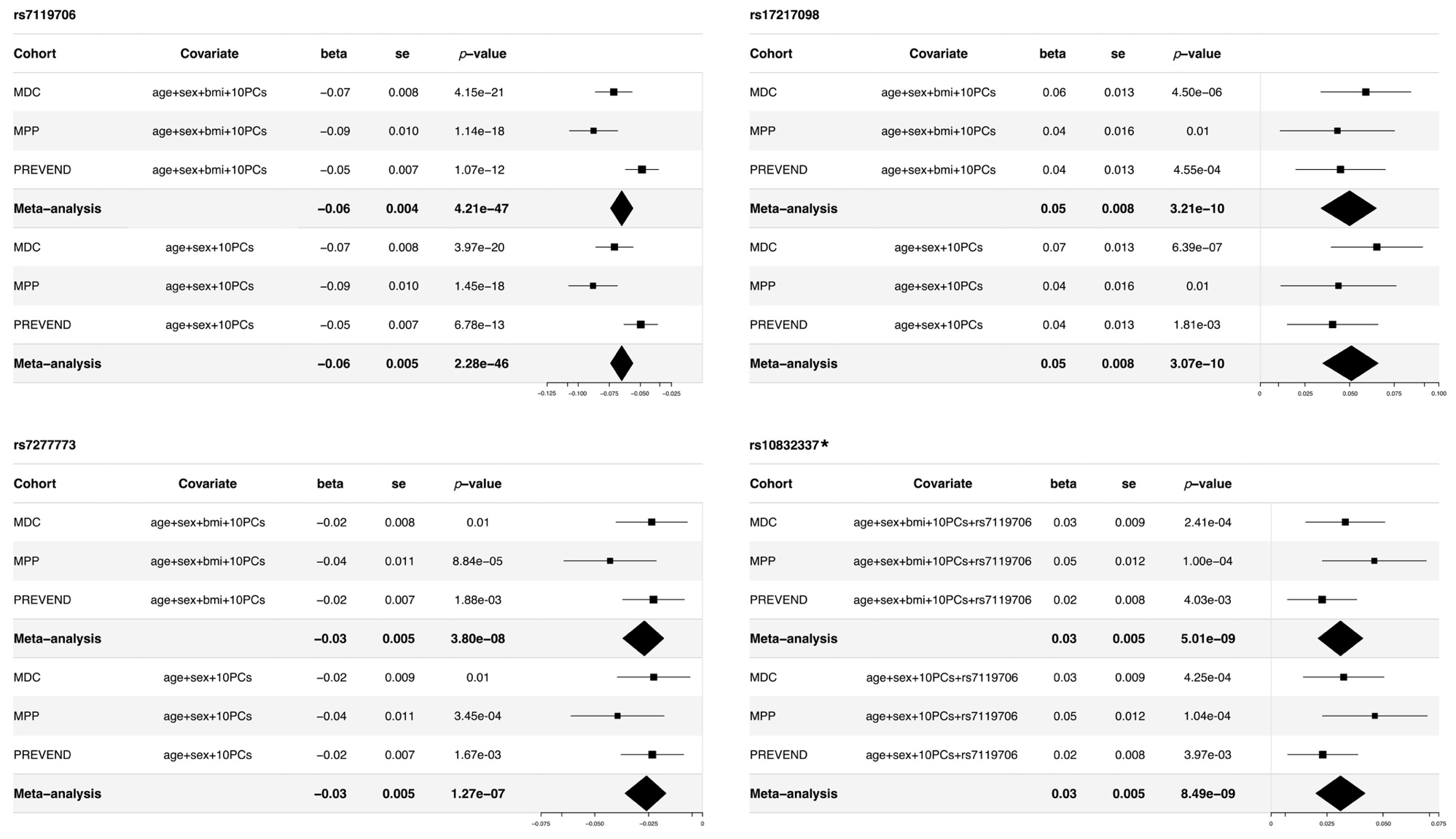
**

**Figure S2.** Effect of the top hits in different cohorts and meta-analysis

*The results of the SNP rs10832337 were the contional analysis results.

**Figure S3.** Regional plots of PCT GWAS and CALCA/CALB pQTLs on chromosome 11.

**Figure S4**. Manhattan plot of multi-trait analysis of GWAS (MTAG) results
